# Impact of Significant Dyads on Dominance Indices in Pigs

**DOI:** 10.3390/ani9060344

**Published:** 2019-06-12

**Authors:** Kathrin Büttner, Irena Czycholl, Katharina Mees, Joachim Krieter

**Affiliations:** Institute of Animal Breeding and Husbandry, Christian-Albrechts-University, Olshausenstr. 40, D-24098 Kiel, Germany; iczycholl@tierzucht.uni-kiel.de (I.C.); katharina.mees@web.de (K.M.); jkrieter@tierzucht.uni-kiel.de (J.K.)

**Keywords:** pig, behavior, significant dyads, dominance index

## Abstract

**Simple Summary:**

Information about the real dominance status of an animal is important to prevent misinterpretations about the social structure in animal groups. Thus, “one fight, won more” must not be interpreted as being dominant. Here, significant dyads help to answer the question of whether one animal is definitely dominant over another, and to clearly evaluate the social hierarchy within a group of animals. In the present study, two calculation methods for the determination of significant dyads in pigs are proposed: on the pen level, i.e., taking all fights in the pen into account, and on the dyad level, i.e., each dyad is considered individually. In three mixing events (weaned piglets, fattening pigs, gilts) the impact of significant dyads on dominance indices was calculated. The number of significant dyads was low but the rank order between the two different methods remained stable. Comparing both of the proposed methods, limits should be calculated on pen level due to the higher flexibility. Thus, significant dyads can be also determined in pens with a low number of fights.

**Abstract:**

Dominance indices are calculated by considering the differences between the number of won and lost fights. Whether these differences show a significant asymmetric outcome or not is neglected. Thus, two calculation methods for the limits of significant dyads are proposed using a sign test based on the differences in won and lost fights, considering all dyadic interactions in the pen (PEN: pen individual limits), and a sign test focusing on each individual dyad (DYAD: dyad individual limits). These were compared to the data set containing all dyadic interactions (ALL). Agonistic interactions in three mixing events were video recorded for two and a half days (weaned piglets) or one and a half days (fattening pigs, gilts). Dominance indices (DI) were calculated for all data sets. Pen/dyad individual limits revealed a small number of significant dyads (weaned piglets: 12.4%/8.8%; fattening pigs: 4.2%/0.6%; gilts: 3.6%/0.4%). Pen individual limits should be selected as they allow adaption of the limits according to the fighting frequency. Spearman rank correlation coefficients of the dominance indices between the data sets were always above 0.7, implying that the rank order remained relatively stable. Information about the impact of significant dyads on sociometric measures is important to prevent misinterpretations about the social structure in animal groups and should be considered in future studies.

## 1. Introduction

According to Makagon et al. [1], the behavior of farm animals both influences and is influenced by the behavior of their pen mates, i.e., an individual’s interactions with others are dependent on the behavior of their pen mates and vice versa [2,3,4,5]. Thus, knowledge about the development and the spread of behavioral patterns within a group (e.g., agonistic interactions, behavioral disorders such as tail biting) is important to understand the social mechanisms behind each behavioral pattern and allows the development, improvement and implementation of prevention and intervention strategies [1].

One example of these behavioral patterns is the establishment of a stable hierarchy within a group of animals after rehousing and mixing. Particularly in pig production, the rehousing and mixing of unacquainted animals is a standard procedure that leads to rank fights, i.e., agonistic interactions, between the animals with a variety of individual pig behaviors ranging from very subtle ritualized gestures to long-lasting overt agonistic interactions with increasing intensity of aggression [6]. There are various parameters that help to describe and analyze social relationships within a group of animals. Sociometric measures are used to characterize dominance relationships and hierarchies within a group of animals. There are different levels which have to be considered. First of all the level of the individual animal, followed by the level of pairwise relationships, the so-called dyads and lastly, the level of the whole group, with regard to all animals in each pen [7].

One basic assumption used to characterize a group of animals with the help of sociometric measures is the definition of dominance described by Drews [8]. Here, dominance is defined as the pattern of repeated agonistic interactions between two individuals, which is characterized by the consistent outcome of the agonistic interactions to the advantage of one individual. According to theoretical studies on dominance, the consistency of the outcome of dyadic interactions should be tested for significance before further sociometric measures are calculated [9,10]. Thus, the assumption that one animal dominates another one in a dyadic relationship if it has won one fight more in a number of agonistic interactions with the same opponent has to be reconsidered as this approach neglects the significant asymmetry of the outcome [7,11], i.e., a dyad is considered as established whenever an animal wins only one agonistic encounter more than it loses [12,13]. Here, if the definition of dominance according to Drews [8] is applied, the significant asymmetry in the outcome of the dyadic interaction between two animals is not taken into account, which may lead to some misinterpretation [7]. According to Langbein and Puppe [7], there is no agreement or even discussion of if, and to what extent dyadic interactions should be tested for a significant asymmetric outcome before including it in further analysis. They suggest considering at least the percentage of dyads that were tested to be significant in order to provide detailed insights into the group structure. This is of particular importance for social groups with a high number of two-way dyads pointing to a high level of bidirectionality. There are only a few studies on farm animals that have included information about significant dyads. They used both empirical measurements [14,15,16] and objective statistical methods [7,11,17,18] to test the asymmetry of the outcome of wins and defeats of dyadic agonistic interactions. Exactly how the exclusion of insignificant dyads might impact the results of sociometric measures has not been clarified in detail until now. 

Thus, in the present study, the impact of significant dyadic interactions on the outcome of sociometric measures was investigated. For this purpose, animals were video recorded for 28 h (weaned piglets) and 17 h (fattening pigs, gilts) directly after rehousing and mixing. For the whole observation period, agonistic interactions were documented. Based on two different calculation methods, significant dyads were determined, which were then compared to the original data set including all dyadic interactions. For all three data sets, the impact of significant dyads on sociometric measures was evaluated. It is hypothesized that depending on the analyzed age groups, different amounts of significant dyads are determined, e.g., younger animals perform more agonistic interactions, which could also be influential for the number of significant dyads. Furthermore, it is hypothesized that limits for significant dyads based on each individual dyad may be too rigid and are not appropriate for animal groups with a low level of overt agonistic interactions. Only if the impact of significant dyads on sociometric measures is known, misinterpretations about the social structure in animal groups can be prevented. Furthermore, quantifying this important aspect of group structure with and without inclusion of significant dyads could help in understanding the formation of specific behavioral patterns and stable dominance relationships. Especially, if the dominance hierarchy is related to managing aggression or behavioral disorders, then reliable indicators are needed.

## 2. Materials and Methods 

### 2.1. Animals and Housing

The study was conducted on the “Hohenschulen” research farm of the Institute of Animal Breeding and Husbandry of the University of Kiel (Germany) from December 2010 until August 2012. The herd consisted of purebred and crossbred animals of the German Landrace and Large White breeds. All males were castrated. The behavior of the animals was analyzed in three repeated rehousing and mixing events: from the farrowing stable to the flatdeck pens, from the flatdeck pens to the fattening stable and from the fattening stable to the arena pen in the breeding stable. In, total, 149 animals could be tracked for the whole period from weaned piglet to gilt. In each rehousing and mixing event, the animals were sorted by the smallest level of familiarity, and weaned piglets and farrowing pigs were additionally sorted by nearly equal body weight. This study was conducted on a pig farm that used conventional practices. After the animals reached slaughter weight, they were slaughtered in a commercial facility. 

#### 2.1.1. Weaned Piglets

After the suckling period of 26 days, the piglets were rehoused and mixed in flatdeck pens. No piglets knew each other from the farrowing unit. In total, there were four compartments with ten pens each. A pen measured 2.05 m × 1.36 m and had a concrete and metal-based floor with no substrate. In each pen, two nipple drinkers were available for ad libitum use of water. The piglets were fed ad libitum with solid pelleted feed. Each pen was equipped with metal chains with a manipulable plastic toy (“Beißmond”, Schulz und Bremer GmbH). Altogether, 829 weaned piglets in 10 consecutive batches every 21 days resulting in 93 pens in total were included in the present study. On average 8.9 ± 0.6 animals were kept in each pen with an average male:female ratio of 1:1.14.

#### 2.1.2. Fattening Pigs

After being in the flatdeck pens for an average age of 70 days, the animals were rehoused in the growing stable. Here, a maximum of two animals were already acquainted with each other from the flatdeck pens. In total, there were five compartments with six pens each. The pens measured 3.25 m × 2.40 m with a half-slatted and half-solid floor. The animals were fed by an automatic mash feeding machine with a commercial diet and had ad libitum access to water provided through nipple drinkers. As enrichment material, metal chains with manipulable plastic pipes and balls were offered. In total, 543 fattening pigs in five consecutive batches every 21 days resulting in 26 pens were included in the present study with an average of 20.9 ± 1.7 animals/pen. The average male:female ratio was 1:1. Animals not used for breeding were slaughtered in a commercial facility after they reached slaughter weight.

#### 2.1.3. Gilts

In the 22nd week of age (with an average of 154 days) gilts were moved from the fattening stable to the arena pen in the breeding stable. Here, a maximum of five animals were already acquainted from the former rehousing and mixing events. The arena pen measured 7.20 m × 5.40 m and had a half-slatted and half-solid floor. The gilts were fed by an automatic mash feeding machine with commercial gilt feed. Water was accessible through nipple drinkers. As enrichment material, metal chains and manipulable plastic balls were offered. In total, 249 gilts selected out of the batches of the fattening pigs were integrated in the breeding stable in the arena pen over 12 consecutive batches each 5 days with an average of 20.8 ± 3.4 animals/pen.

#### 2.1.4. Ethical Statement

All pigs were normally farmed animals. The disturbance to the animals was kept to a minimum as the main focus of the study was on video recordings. The authors declare that the experiments were carried out strictly following international animal welfare guidelines thereby adhering to the “German Animal Welfare Act” (German designation: TierSchG), the “German Order for the Protection of Animals used for Experimental Purposes and other Scientific Purposes (German designation: TierSchVersV), and the “German Order for the Protection of Production Animals used for Farming Purposes and other Animals kept for the Production of Animal Products” (German designation: TierSchNutztV) were applied. No pain, suffering or injury was inflicted on the animals during the experiment.

### 2.2. Video Observation

The behavior of the animals was recorded with the help of video cameras mounted on the ceiling in order to achieve a complete overview of each pen. For each pen, one camera (SANTEC day/night color camera VTC-249IRP/W, SANTEC BW AG, Ahrensburg, Germany) was used. The animals had full artificial lighting between 06.00 and 19.00 h and the by law required window area for daylight (3% of the housing floor area). The light intensity was not reduced during the mixing procedure.

Stukenborg et al. [19] described a decline in fighting behavior overnight. Thus, video observation was paused from 18:00 h to 07:00 h. Due to the fact that older animals showed less agonistic interactions [11,20], for weaned piglets, two and a half days and for fattening pigs and gilts one and a half days of behavioral data were directly analyzed after rehousing and mixing (rehousing started at 12:00 h). In total, for weaned piglets the period used for analyses was 28 h (day 1: 12:00 h to 18:00 h; day 2: 07:00 h to 18:00 h; day 3: 07:00 h to 18:00 h) and 17 h was used for fattening pigs and gilts (day 1: 12:00 h to 18:00 h; day 2: 07:00 h to 18:00 h). Video data were analyzed using the HeiTelPlayer software (Xtralis Headquarter D-A-CH, HeiTel Digital Video GmbH, Kiel, Germany).

### 2.3. Agonistic Interactions

Directly after rehousing and mixing, the occurrence and the outcome of the agonistic interactions were recorded from the videos by three observers for weaned piglets (i.e., the recorded video footage was divided into three equally sized portions, which were randomly assigned to and then analyzed by one of the three observers who had been trained at the beginning of the video analysis), and to one observer for fattening pigs and gilts. For the training, unknown video sequences (i.e., the observers have not seen these video sequences before), which were not part of the present study were used in order to practice the definition and the detection of agonistic behavior. After the training the inter-observer reliability was above 90%. Directly before the mixing event, all animals were marked with individual signs on their backs using a livestock marking spray (“Viehzeichenspray BEG”, BEG Schulze Bremer GmbH, Dülmen, Germany) so that the animals involved in an agonistic interaction could clearly be identified. 

According to Langbein and Puppe [7], an agonistic interaction was defined as a fight or displacement with physical contact, which was initiated by one pig, and included aggressive behavioral elements, followed by submissive behavior from the opponent. The following aggressive behavioral patterns were recorded: parallel or inverse pressing, head to body knock, head to head knock, biting, and physical displacement [7,19,21,22]. A fight started when a pig showed one of the above behavioral patterns and it lasted for more than 1 s. The fight ended when one of the encounters showed a submissive behavior (e.g., turning away, fleeing) and when the pigs were separated for at least 5 s after the fight [23]. The animal that showed the submissive behavior was recorded as the loser of the agonistic interaction. For each agonistic interaction the initiator, the receiver, the winner, the loser as well as the duration of the fight were documented. For further analysis, only fights between two animals with a clear initiator or receiver and with a clear winner and loser were used.

### 2.4. Calculation of Significant Dyads

A dominant relationship between two individuals is characterized by the consistent outcome of the agonistic interactions to the advantage of one individual [8]. Thus, the significant asymmetric outcome of dyadic interactions has to be tested before further sociometric measures are calculated [9,10]. Therefore, in the present study, two different calculations for the limits of significant dyads were carried out, considering pen individual or dyad individual limits, which are described in detail below.

#### 2.4.1. Pen Individual Limits for a Significant Asymmetric Outcome

The pen individual limits for a significant asymmetric outcome were calculated with the help of a one-sided sign test, which has the null hypothesis that the median of the differences (animal with higher number of fights won—animal with lower number of fights won) in each dyad is zero. This test includes the data of every dyad in the pen and provides a 95% confidence interval, which is used to obtain pen individual limits for the significant asymmetric outcome of the dyadic interactions. For the calculation of the one-sided sign test, the R package BSDA [24] was used.

#### 2.4.2. Dyad Individual Limits for a Significant Asymmetric Outcome

The dyad individual limits were calculated according to the methodology used in Langbein and Puppe [7]. Here, the significance of the asymmetry of each single dyadic interaction was explicitly tested by a sign test [25,26]. In contrast to Langbein and Puppe [7], in the present study a one-sided sign test was used instead of a two-sided test. This procedure was chosen due to the fact that the question which had to be answered was whether the animal had won significantly more fights, so the test statistic has to be one-sided. Performing this one-sided sign test led to the conclusion that at least five agonistic interactions with a strictly unidirectional outcome were required to reach significance (*p* < 0.05), i.e., 5 wins vs. 0 defeats. In Table 1, an extract of the critical values x_max_ for the one-sided sign test at an α level of 0.05 is presented.

A significant asymmetric outcome between two animals in the number of won or lost fights exists, if the observed value x (which is the number of won fights of the animal with the lower number of won fights) is smaller than the critical value x_max_ at a given number of agonistic interactions n. 

### 2.5. Data Sets

As described above, for all three considered age groups (weaned piglets, fattening pigs, gilts) only agonistic interactions between two animals with a clear initiator and a clear receiver as well as a clear winner and a clear loser were used for further analysis. These data sets are referred to as ALL in the rest of the manuscript. On the basis of this data set, significant dyads were calculated for the two described calculation methods. The data set PEN contains the significant dyads calculated with the help of the pen individual limits for a significant asymmetric outcome. The data set DYAD contains the significant dyads calculated with the help of the dyad individual limits for a significant asymmetric outcome. Table 2 illustrates the basic information for the three used data sets. Due to the low number of pens involved in the data sets DYAD for fattening pigs and gilts; i.e., only three pens for fattening pigs and one pen for gilts contained significant dyads according to the dyad individual limits; only the comparison between the data sets ALL and PEN are shown for these age groups in the results section. Furthermore, in order to compare the three age groups, the observation time of 17 h (end of video observation at day 2) for weaned piglets was also included in Table 2.

### 2.6. Dyadic Interactions and Number of Fights

Sociometric measures are used to describe the dominance relationships at different levels of social organizations. The dyadic level is distinguished by: unknown dyads, i.e., two animals that never show an agonistic interaction with each other; one-way dyads, i.e., one animal always wins over the other one; two-way dyads, i.e., dyads in which each animal has both wins and defeats; and tied dyads, i.e., both animals have the same number of wins and defeats [7]. Furthermore, the percentage of significant dyads with a significant asymmetric outcome was calculated with two different calculation methods (see Section 2.4). 

Significant differences in the dyadic categories between the three used data sets as well as the overall effect of the age group (weaned piglets, fattening pigs, gilts) on the sum of the agonistic interactions for each animal during 17 h of video observation was estimated with nonparametric Kruskal-Wallis tests using the statistical software package SAS 9.4 [27]. To test pairwise differences between the age groups, subsequent Dunn’s post-hoc tests were carried out.

### 2.7. Dominance Index

In the present study, a dominance index (DI) for each animal was calculated following the formula used in Borberg and Hoy [28] and Fels et al. [29]. The formula includes the number of won fights (wins) and lost fights (defeats), the number of pen mates that had won against the pig (P_won_) or lost (P_lost_) as well as the group size (n).

DI = [(wins × P_won_) − (defeats × P_lost_)]/[(wins + defeats) × (n − 1)] (1)

DIs can only be calculated for animals that were involved in at least one agonistic interaction. DIs range between −1 (absolute submissive animal) to 1 (absolute dominant animal).

In order to evaluate the temporal development of the dominance indices, they were calculated using increasing time window lengths over the whole observation period, i.e., the first time window length comprised the observation times: day 1: 12:00 h until day 1: 13:00 h (includes 1 h), the length of the second time window comprised the observation times: day 1: 12:00 h until day 1: 14:00 h (includes 2 h), and so on until the whole observation period from day 1: 12:00 h until day 3: 18:00 h (includes 28 h) for weaned piglets or until day 2: 18:00 h (includes 17 h) for fattening pigs and gilts was covered.

In order to estimate the effect of the exclusion of dyads with an insignificant asymmetric outcome of the dyadic interaction, for each time window, a Spearman rank correlation using the statistical software package SAS 9.4 [27] was carried out considering the dominance indices of all three used data sets (ALL, PEN, DYAD).

## 3. Results

### 3.1. Dyadic Interactions and Number of Fights

For weaned piglets and considering 28 h of video observation, on average 15.2% or 13.3% of the dyads showed a significant asymmetric outcome for the calculation according to pen (data set PEN) or dyad individual limits (data set DYAD), respectively. Considering only 17 h of video observation in order to enable a comparison between the three observed age groups, the percentages decreased to 12.4% or 8.8%, respectively. For fattening pigs and gilts, an even lower number of significant dyads with 4.2% and 3.6% for pen individual limits (data set PEN) and 0.6% and 0.4% for dyad individual limits (data set DYAD) were obtained. These findings are in accordance with the average number of agonistic interactions per animal in the three observed age groups considering all dyadic interactions, i.e., weaned piglets, with 12.3 fights/animal fought significantly more compared to fattening pigs with 5.9 fights/animal and gilts with 5.3 fights/animal (*p* < 0.05). Due to the low number of observations in the data set DYAD for fattening pigs and gilts (see Table 2), the data set DYAD was excluded for these age groups from further analyses. The means of all calculated sociometric measures on the dyadic level are illustrated in Table 3. Comparing the three used data sets within each age group, weaned piglets had the largest increase with about 50% of unknown dyads from the data set ALL compared to both data sets PEN and DYAD. For fattening pigs and gilts, the differences between the data sets ALL and PEN were not this obvious as the data set ALL already had higher percentages of unknown dyads with about 80% in fattening pigs and gilts.

### 3.2. Dominance Index

#### 3.2.1. Frequency Distribution of the Dominance Indices

**Figure 1:**
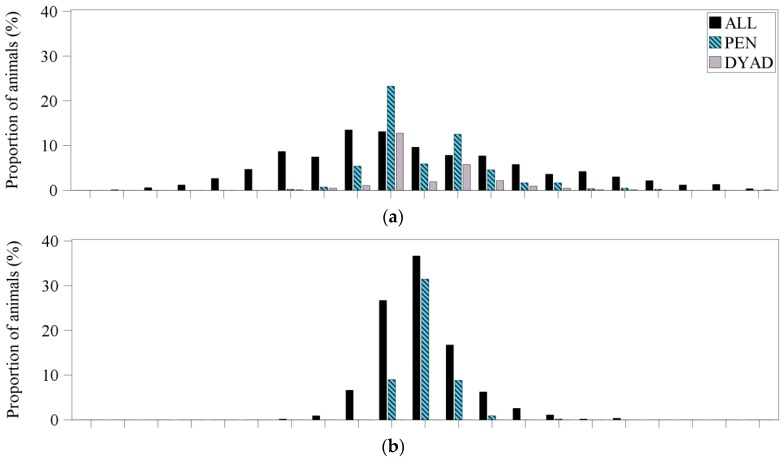

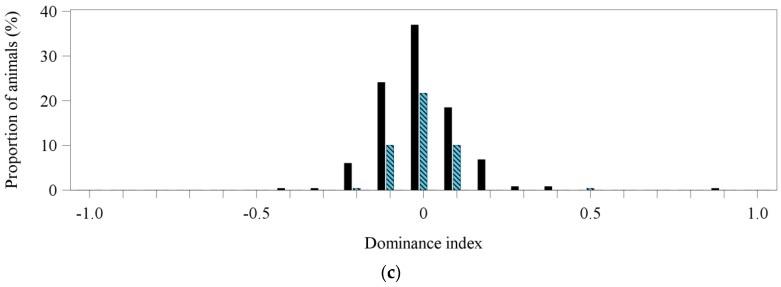
Frequency distribution of the dominance indices for all three age groups ((**a**) weaned piglets, (**b**) fattening pigs, (**c**) gilts) after 17 h (end of video observation of day 2) of video observation and the three used data sets (ALL: including all dyadic interactions; PEN: including only dyads with a significant asymmetric outcome according to pen individual limits; DYAD: including only dyads with a significant asymmetric outcome according to dyad individual limits).

Figure 1 illustrates the frequency distribution of the dominance indices for the three observed age groups (weaned piglets, fattening pigs, gilts) and the three used data sets (ALL, PEN, DYAD) after 17 h of video observation. Video observation for 17 h was used in order to enable a comparison between the three observed age groups. After the first 6 h of video observation after mixing, the distribution of the dominance indices differed only slightly, so, Figure 1 gives a representative image of the dominance indices. This holds for all age groups and data sets analyzed.

#### 3.2.2. Weaned Piglets

Considering the data set ALL, weaned piglets revealed a large variation in dominance indices ranging from −0.9 to 1 (Figure 1a). No dominance index could be calculated for only 8 animals (1%). In contrast, considering the data set PEN, weaned piglets showed a lower variation ranging from −0.4 to 0.7 with the majority of animals receiving a dominance index of −0.1. Here, for 355 animals (43%) no dominance index could be calculated. The frequency distribution of the dominance indices in the data set DYAD displayed a similar shape to the data set PEN. However, in the data set DYAD, one animal was absolutely dominant with a dominance index of 1. In this data set, for 612 animals (74%) no dominance index could be calculated. The frequency distribution of the dominance indices for weaned piglets after 28 h of video observations for all three used data sets showed a similar distribution to the 17 h of video observations illustrated in Figure 1a.

#### 3.2.3. Fattening Pigs

Independent of the used data set, fattening pigs showed a much narrower distribution compared to weaned piglets (Figure 1b). For the data set ALL, the dominance indices ranged from −0.4 to 0.6 with the majority of animals displaying a dominance index of 0. Here, no dominance index could be obtained for only 9 animals (2%). For the data set PEN, the variation was even smaller with values between −0.1 and 0.4. Here, for about 50% of the animals no dominance index could be calculated.

#### 3.2.4. Gilts

Considering the data set ALL, the majority of gilts revealed a dominance index between −0.4 and 0.4, only one gilt had a dominance index of 0.9 (Figure 1c). For 12 gilts (5%) no dominance index could be obtained. Considering the data set PEN, no dominance index could be calculated for 143 gilts (57%). The rest ranged between −0.2 and 0.1 with one outlier with a dominance index of 0.5.

#### 3.2.5. Relationship of the Dominance Indices between the Three Used Data Sets

**Figure 2:**
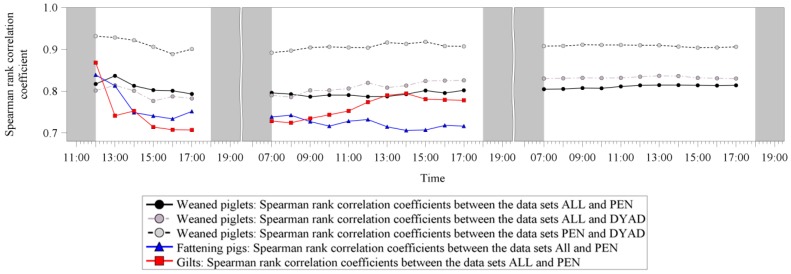
Spearman rank correlation coefficients for the calculated dominance indices for all three age groups (weaned piglets, fattening pigs, gilts) between the three used data sets (ALL: including all dyadic interactions; PEN: including only dyads with a significant asymmetric outcome according to pen individual limits; DYAD: including only dyads with a significant asymmetric outcome according to dyad individual limits). Grey filled areas on the x-axis illustrate times without video observation. For fattening pigs and gilts only one and a half days of video observation were carried out.

Figure 2 illustrates the Spearman rank correlation coefficients for the calculated dominance indices between the three used data sets. For weaned piglets, the r_s_ values between the data set ALL and the data sets PEN and DYAD ranged between 0.78 and 0.84 at a constant level. The r_s_ values between the data sets PEN and DYAD were higher, ranging from 0.89 to 0.93. For fattening pigs and gilts, a clear drop in the r_s_ values after the first 3 h could be detected. However, here also the r_s_ values never dropped under a value of 0.70. Comparing the three different age groups, weaned piglets revealed slightly higher r_s_ values between the dominance indices for the data sets ALL and PEN compared to fattening pigs and gilts.

## 4. Discussion

In the literature [7,9,10,11] it is claimed that the consistency of the outcome of dyadic interactions has to be tested for significance before they are used for further calculations in order to match the dominance definition proposed by Drews [8]. Thus, due to the fact that uncertainties about the correct methodology also exist, in the present study, two different calculation methods for the determination of significant dyads were proposed and their impact on dominance indices was evaluated.

### 4.1. Dyadic Interactions and Number of Fights

The results of the different categories of dyadic interactions showed that the percentage of unknown dyads for the data set ALL was higher and the percentage of significant dyads was lower compared to other studies [7,11]. The data sets PEN and DYAD revealed an even higher number of unknown dyads compared to the data set ALL. Puppe et al. [11] analyzed the agonistic interactions of pigs in three age groups directly after rehousing. Here, weaned piglets (age: 28 days) and fattening pigs (age: 80 days) had with 10% and 3% significantly lower amounts of unknown dyads compared to multiparous sows with 22%. Hence, these unknown dyads were clearly lower in number compared to the present study. In Langbein and Puppe [7], only one group of pigs was observed directly after weaning. Here, the number of unknown dyads was even lower with 4%. However, although the group sizes of weaned piglets in the study of Puppe et al. [11] were comparable to the present study, the group sizes for fattening pigs and gilts were only half as large as the groups in the present study, which could be one explanation for the higher number of unknown dyads [30]. According to Marchant-Forde and Marchant-Forde [31] larger group size in pigs revealed less agonistic interactions after mixing compared to smaller group size. These findings are also confirmed by other studies [32,33,34]. However, also the size of the pens could be an explanation for this relationship. More space available (i.e., lower stocking densities or constant stocking densities in combination with larger group sizes) provides more means of escape for subordinate animals and thus, less agonistic interactions [35,36]. Additionally, according to Shizuka and McDonald [37] dyadic interactions may fail to occur, i.e., an increase in unknown dyads, for various reasons. Some animals may actively avoid agonistic interactions due to the fact that the costs of a fight are too high (e.g., probability of injuries, too high energy costs), the possible benefits of a fight are too low, or the rank order can be clarified without the performance of overt agonistic interactions (e.g., new coping strategies [38,39]). Also, the level of familiarity may impact the number of agonistic interactions. According to Puppe [22] and Arey and Franklin [40], familiar animals showed less agonistic interactions. Furthermore, the fact that some agonistic interactions may be missed by the observers is one possible explanation for unknown dyads [37] and should not be neglected. 

In both studies by Langbein and Puppe [7] and Puppe et al. [11], the amount of significant dyads, which were calculated according to a two-sided sign test [25,26], with about 35—38% for weaned piglets, 54% for fattening pigs and 23% for sows, which is clearly higher compared to the results of the present study. Although the calculation methods for significant dyads differed slightly between the present study and the two studies of Langbein and Puppe [7] and Puppe et al. [11], i.e., the two-sided sign test has lower limits for classification as a dyad with a significant asymmetric outcome, this cannot be the only explanation for the extreme difference in the amount of significant dyads. Here, the lower level of agonistic interactions as well as the higher number of unknown dyads may be a better reason for the low number of significant dyads. Also, the study of Puppe et al. [11] stated highly negative Spearman rank correlation coefficients between unknown and significant dyads with −0.91 for weaned piglets, −0.74 for fattening pigs and −0.66 for sows.

The results of the present study revealed with the high standard deviation for the number of different categories of dyadic interactions, that there is a huge variation in the behavioral patterns of each single animal group which has to be taken into account. Therefore, it is questionable if the application of rigid boundaries for significant dyads is an appropriate method to describe behavioral patterns for a specific group of animals, which is done using the dyad individual limits for a significant asymmetric outcome. Other influencing factors such as animal health, season, housing, weight, recent social experiences, group composition and management [18,22,33,34] should be included in the analyses in order to get a clearer picture about the formation of specific behavioral patterns and the variation between individual pens. Martin et al. [18] also stated that the outcome of agonistic interactions is determined by many different factors and that dominance appears as a result of more or less significant asymmetries in a set of individual characteristics and environmental impacts. One important aspect that should also be considered in further analysis is the determination of the personality of each animal. In particular, the personality trait aggression in the case of agonistic interactions and dominance relationships is of special importance [41], and this varies considerably between animals [42]. The different levels of aggressiveness of the animals can be measured with the resident-intruder test. In this test, a resident pig is kept in its home pen and is confronted there with an approximately 20% smaller and unfamiliar intruder pig, which typically leads to an agonistic interaction, i.e., the resident pig attacks the intruder pig. Here, the latency time until the first attack is recorded, with lower values indicating a more aggressive response. Thus, not only the pig’s own fighting ability might influence the contest behavior but also the personality trait aggressiveness. Here, in groups of animals with a low level of aggressiveness, agonistic interactions might involve more ritualized displays or non-damaging behavior than damaging behavior [41]. This again would be beneficial for animal welfare because of the lower number of injuries due to overt agonistic interactions. However, less overt agonistic interactions are also more complicated for reliable recording.

### 4.2. Dominance Index 

Due to the fact that only for weaned piglets the percentage of significant dyads according to dyad individual limits were high enough for statistical analyses, a comparison between the data sets PEN and DYAD was only possible for this age group. Here, the results illustrate that the parameters in the two data sets PEN and DYAD were highly correlated. However, for further analysis it has to be determined which of the two proposed calculation methods is preferred. In the present study, a huge variation in the number of agonistic interactions between the single pens could be observed, which raises the question whether dyad individual limits for a significant asymmetric outcome are appropriate. In pens with a low number of agonistic interactions, one can assume that the social hierarchy within this group is relatively stable and the rank order is already established [43]. In the present study, the fattening pigs and gilts demonstrated a much lower frequency of agonistic interactions. This might be due to their increasing age or due to the rising level of familiarity with each rehousing event. Other studies have confirmed the findings that younger animals had more frequent and intense agonistic interactions compared to older animals [11,20]. Also the level of familiarity plays an important role in the establishment of a rank order, i.e., the higher the level of familiarity between the animals, the faster the rank order is established [22,40]. If the dyad individual limits of significant dyads are applied to pens with only a low number of agonistic interactions, the percentage of significant dyads becomes so low that these results cannot be used for the calculation of further sociometric measures. Pens with an average low number of agonistic interactions have a lower possibility for significant dyads due to the fact that at least five agonistic interactions between two individuals, which resulted in a one-way dyad had to occur in order to be classified as dyadic interaction with a significant asymmetric outcome according to the dyad individual limits. In contrast, the pen individual limits take all differences between each dyad present in the group of animals into account to obtain pen individual limits for the testing for significance. Here, the limits are lower compared to the dyad individual limits, which allow more dyads to become significant. Also, in order to describe the social hierarchy in low fighting groups the pen individual limits are preferred over the dyad individual limits.

### 4.3. Other Possibilities to Test for Significant Dyadic Interactions

In the literature, there are only a few studies on farm animals that include information about significant dyads. In the studies of Langbein and Puppe [7], Puppe et al. [11] and Puppe and Tuchscherer [17], a two-sided sign test to test for a significant asymmetric outcome of each individual dyad was carried out, which has slightly lower limits for a significant asymmetric outcome compared to the one-sided sign test proposed in the present study. In Martin et al. [18], the dominance relationships of hens were analyzed. Here, a hen was said to be dominant when an agonistic asymmetry between two animals could be recorded for six consecutive agonistic interactions. Hunter et al. [14] defined a sow as being dominant over another sow if at least two agonistic interactions with the same outcome were present, and whenever reversals were present a ratio of 4:1 was necessary to assume the dominance of one sow. Araba and Crowell-Davis [15] defined a foal to be dominant over another foal if it won five agonistic interactions and lost none. However, if reversals occur, then in addition to the basic five wins the foal had to win another two agonistic interactions for each defeat in order to be classified as dominant. To what extent these different approaches for the determination of significant dyads influence the outcome of sociometric measures was not evaluated, which should be part of further studies. Also, an alternative to the two proposed calculation methods is recommended.

### 4.4. Implications for Further Studies and Practical Implementation

With the determination of dyadic interactions with a significant asymmetric outcome, i.e., the determination of real dominant-subordinate pairs, and the information about the number of insignificant dyads, i.e., dyadic interactions without a clear dominant or subordinate animal, can be used to enhance understanding about group formation. A high number of insignificant dyads indicates a high number of animals with a similar or no clear rank position. If this number is high, this group composition can lead to increased agonistic interactions with higher stress levels, especially for the animals with an uncertain rank position within the group [44,45]. However, in the present study, only physically aggressive behaviors were recorded and used for the determination of significant dyads. Thus, it cannot be concluded that all insignificant dyads are animals with an uncertain rank position within the group or if rank order was clarified by other behaviors such as ritualized displays or threats, which were not recorded in the present study. This became even more obvious in the older age groups of fattening pigs and gilts, which have already acquired other coping strategies without overt agonistic interactions in order to establish a social hierarchy. Therefore, in further studies, ritualized displays or threats should be also included in the ethogram considering the fact that these behaviors are way more complicated to determine and record compared to overt agonistic interactions. Also, the inclusion of behavioral tests such as the resident-intruder test may enhance the results of the present study. Combining information about significant dyads with the personality of each pig obtained in the resident-intruder test may be used for the breeding of less aggressive animals who clarify their rank position with less damaging behavior. According to the dominance definition of Drews [8] obtaining the real rank position is also needed in order to correlate the social hierarchy within a group to behavioral disorders, such as tail biting. Statements about the impact of the rank position on being a biter or a victim of tail biting can only be made if the real dominance hierarchy is known. 

## 5. Conclusions

In the present study, the impact of two different calculation methods concerning the significant asymmetric outcome of dyadic interactions on the outcome of sociometric measures was evaluated. Both calculation methods revealed only a small number of dyads with a significant asymmetric outcome for the pen and dyad individual limits (weaned piglets: 12.4% or 8.8%; fattening pigs: 4.2% or 0.6%; gilts: 3.6% or 0.4%). Because the dyad individual limits are based on rigid boundaries, pen individual limits for significant dyads should be selected as they allow for an adaption of the limits according to the number of agonistic interactions within each pen. Thus, in animal groups with a low amount of agonistic interactions, significant dyads can also be determined. Although the percentages of significant dyads were low in all three observed age groups, the Spearman rank correlation coefficients of the dominance indices between the three used data sets were always above 0.7, implying that the rank order remained relatively stable. Due to the fact that there is yet no standardized way for the determination of dyadic interactions with a significant asymmetric outcome, future studies should include a detailed description of the definition of significant dyads. Furthermore, all calculations should be carried out using the data sets containing all dyadic interactions and containing only significant dyads in order to evaluate the impact of significant dyads on the outcome of the analyses. Overall, this study provides insights into the importance of the calculation of significant dyads and its impact on sociometric measures. Thus, in future studies the reliability and accuracy of these sociometric measures can be better evaluated.

## Figures and Tables

**Table 1:** Extract of critical values x_max_ for the one-sided sign test at an α level of 0.05 for a given number of agonistic interactions n. A significant asymmetric outcome between two animals in the number of won or lost fights exists, if the observed value x (which is the number of won fights of the animal with the lower number of won fights) is smaller than the critical value x_max_ at a given number of agonistic interactions n, i.e., if x < x_max_, the dyad has a significant asymmetric outcome according to the dyad individual limits.

n	x_max_	n	x_max_	n	x_max_
5	1	12	3	19	6
6	1	13	4	20	6
7	1	14	4	21	7
8	2	15	4	22	7
9	2	16	5	23	8
10	2	17	5	24	8
11	3	18	6	25	8

**Table 2:** Basic information about the three used data sets (ALL: including all dyadic interactions; PEN: including only dyads with a significant asymmetric outcome according to pen individual limits; DYAD: including only dyads with a significant asymmetric outcome according to dyad individual limits) for the three observed age groups (weaned piglets, fattening pigs, gilts).

	Data Set		
	ALL	PEN	DYAD
**Weaned piglets (28 h of video observation)**			
Number of pens	93	92	61
Number of animals	829	820	548
Mean (±SD) number of animals/pen	8.9 ± 0.6	8.9 ± 0.6	9.0 ± 0.5
Number of agonistic interactions	7620	3351	2495
Mean (±SD) number of agonistic interactions/pen	81.9 ± 63.6	36.4 ± 37.0	40.9 ± 44.5
**Weaned piglets (17 h of video observation)**			
Number of pens	93	90	53
Number of animals	829	806	474
Mean (±SD) number of animals/pen	8.9 ± 0.6	9.0 ± 0.5	8.9 ± 0.5
Number of agonistic interactions	5088	2151	1228
Mean (±SD) number of agonistic interactions/pen	54.7 ± 36.2	23.9 ± 21.7	23.2 ± 23.3
**Fattening pigs (17 h of video observation)**			
Number of pens	26	26	3
Number of animals	543	543	60
Mean (±SD) number of animals/pen	20.9 ± 1.7	20.9 ± 1.7	20 ± 2.7
Number of agonistic interactions	1611	552	19
Mean (±SD) number of agonistic interactions/pen	62.0 ± 13.7	21.2 ± 10.1	6.3 ± 1.5
**Gilts (17 h of video observation)**			
Number of pens	12	12	1
Number of animals	249	249	22
Mean (±SD) number of animals/pen	20.8 ± 3.4	20.8 ± 3.4	22
Number of agonistic interactions	665	209	5
Mean (±SD) number of agonistic interactions/pen	55.4 ± 19.0	17.4 ± 11.6	5

**Table 3:** Descriptive statistics (mean ± standard deviation) of the different categories of dyadic interactions for all age groups (weaned piglets, fattening pigs, gilts) and the three used data sets (ALL: including all dyadic interactions; PEN: including only dyads with a significant asymmetric outcome according to pen individual limits; DYAD: including only dyads with a significant asymmetric outcome according to dyad individual limits).

	Data Set		
	ALL	PEN	DYAD
**Weaned piglets (28 h of video observation)**			
Unknown dyads (%)	31.7 ± 16.8 ^a^	84.8 ± 7.7 ^b^	86.7 ± 11.2 ^b^
One-way dyads (%)	51.4 ± 11.0 ^a^	11.9 ± 5.6 ^b^	10.7 ± 8.3 ^b^
Two-way dyads (%)	13.0 ± 12.8 ^a^	3.3 ± 4.2 ^b^	2.6 ± 4.0 ^b^
Tied dyads (%)	13.0 ± 12.8 ^a^	3.3 ± 4.2 ^b^	2.6 ± 4.0 ^b^
**Weaned piglets (17 h of video observation)**			
Unknown dyads (%)	40.9 ± 16.7 ^a^	85.2 ± 7.5 ^b^	87.2 ± 10.7 ^b^
One-way dyads (%)	48.2 ± 11.9 ^a^	12.8 ± 5.7 ^b^	10.7 ± 8.2 ^b^
Two-way dyads (%)	7.3 ± 7.8 ^a^	2.4 ± 3.4 ^b^	2.0 ± 3.8 ^b^
Tied dyads (%)	7.3 ± 7.8 ^a^	2.4 ± 3.4 ^b^	2.0 ± 3.8 ^b^
**Fattening pigs (17 h of video observation)**			
Unknown dyads (%)	78.6 ± 4.3 ^a^	95.8 ± 2.0 ^b^	-
One-way dyads (%)	19.6 ± 4.0 ^a^	4.0 ± 1.8 ^b^	-
Two-way dyads (%)	0.7 ± 0.8 ^a^	0.2 ± 0.4 ^b^	-
Tied dyads (%)	0.7 ± 0.8 ^a^	0.2 ± 0.4 ^b^	-
**Gilts (17 h of video observation)**			
Unknown dyads (%)	81.2 ± 6.6 ^a^	96.4 ± 2.9 ^b^	-
One-way dyads (%)	16.3 ± 6.6 ^a^	3.4 ± 2.9 ^b^	-
Two-way dyads (%)	0.9 ± 0.7 ^a^	0.2 ± 0.2 ^b^	-
Tied dyads (%)	0.9 ± 0.7 ^a^	0.2 ± 0.2 ^b^	-

^a,b^ Significant differences (*p* < 0.05) between the data sets are indicated by different letters.

## References

[1] Makagon M.M., McCowan B., Mench J.A. (2012). How can social network analysis contribute to social behavior research in applied ethology?. Appl. Anim. Behav. Sci..

[2] Wasserman S., Faust K. (1994). Social Network Analysis: Methods and Applications.

[3] Croft D.P., James R., Ward A.J.W., Botham M.S., Mawdsley D., Krause J. (2005). Assortative interactions and social networks in fish. Oecologia.

[4] Krause J., Croft D.P., James R. (2007). Social network theory in the behavioural sciences: Potential applications. Behav. Ecol. Sociobiol..

[5] Madden J.R., Drewe J.A., Pearce G.P., Clutton-Brock T.H. (2009). The social network structure of a wild meerkat population: 2. Intragroup interactions. Behav. Ecol. Sociobiol..

[6] Camerlink I., Turner S.P., Farish M., Arnott G. (2017). The influence of experience on contest assessment strategies. Sci. Rep..

[7] Langbein J., Puppe B. (2004). Analysing dominance relationships by sociometric methods—A plea for a more standardised and precise approach in farm animals. Appl. Anim. Behav. Sci..

[8] Drews C. (1993). The Concept and Definition of Dominance in Animal Behaviour. Behaviour.

[9] Boyd R., Silk J.B. (1983). A method for assigning cardinal dominance ranks. Anim. Behav..

[10] Lehner P.N. (1998). Handbook of Ethological Methods.

[11] Puppe B., Langbein J., Bauer J., Hoy S. (2008). A comparative view on social hierarchy formation at different stages of pig production using sociometric measures. Livest. Sci..

[12] Appleby M.C. (1983). The probability of linearity in hierarchies. Anim. Behav..

[13] De Vries H. (1995). An improved test of linearity in dominance hierarchies containing unknown or tied relationships. Anim. Behav..

[14] Hunter E.J., Broom D.M., Edwards S.A., Sibly R.M. (1988). Social hierarchy and feeder access in a group of 20 sows using a computer-controlled feeder. Anim. Prod..

[15] Araba B.D., Crowell-Davis S.L. (1994). Dominance relationships and aggression of foals (*Equus calballus*). Appl. Anim. Behav. Sci..

[16] Côté S.D. (2000). Determining Social Rank in Ungulates: A Comparison of Aggressive Interactions Recorded at a Bait Site under Natural Conditions. Ethology.

[17] Puppe B., Tuchscherer M. (1994). Soziale Organisationsstrukturen beim intensiv gehaltenen Schwein: 3. Mitteilung: Ethologische Untersuchungen zur Rangordnung. Arch. Tierz..

[18] Martin F., Beaugrand J.P., Laguë P.C. (1997). The role of hen’s weight and recent experience on dyadic conflict outcome. Behav. Process..

[19] Stukenborg A., Traulsen I., Puppe B., Presuhn U., Krieter J. (2011). Agonistic behaviour after mixing in pigs under commercial farm conditions. Appl. Anim. Behav. Sci..

[20] Jensen P. (1982). An analysis of agonistic interaction patterns in group-housed dry sows—Aggression regulation through an “avoidance order”. Appl. Anim. Ethol..

[21] McGlone J.J. (1985). A quantitative ethogram of aggressive and submissive behaviors in recently regrouped pigs. J. Anim. Sci..

[22] Puppe B. (1998). Effects of familiarity and relatedness on agonistic pair relationships in newly mixed domestic pigs. Appl. Anim. Behav. Sci..

[23] Samarakone T.S., Gonyou H.W. (2009). Domestic pigs alter their social strategy in response to social group size. Appl. Anim. Behav. Sci..

[24] Arnholt A.T., Evans B. (2017). BSDA: Basic Statistics and Data Analysis. https://rdrr.io/cran/BSDA/.

[25] Dixon W.J., Mood A.M. (1946). The Statistical Sign Test. J. Am. Stat. Assoc..

[26] Röhr M., Lohse H., Ludwig R. (1983). Statistik für Soziologen, Pädagogen, Psychologen und Mediziner. Band 2-Statistische Verfahren.

[27] SAS^®^ Institute Inc. (2013). User’s Guide (Release 9.4).

[28] Borberg C., Hoy S. (2009). Mixing of sows with or without the presence of a boar. Livest. Sci..

[29] Fels M., Hoy S., Hartung J. (2012). Influence of origin litter on social rank, agonistic behaviour and growth performance of piglets after weaning. Appl. Anim. Behav. Sci..

[30] Klass K., Cords M. (2011). Effect of unknown relationships on linearity, steepness and rank ordering of dominance hierarchies: Simulation studies based on data from wild monkeys. Behav. Process..

[31] Marchant-Forde J.N., Marchant-Forde R.M. (2005). Minimizing inter-pig aggression during mixing. Pig News Info..

[32] Turner S.P., Horgan G.W., Edwards S.A. (2001). Effect of social group size on aggressive behaviour between unacquainted domestic pigs. Appl. Anim. Behav. Sci..

[33] Nielsen B.L., Lawrence A.B., Whittemore C.T. (1995). Effect of group size on feeding behaviour, social behaviour, and performance of growing pigs using single-space feeders. Livest. Prod. Sci..

[34] Andersen I.L., Nævdal E., Bakken M., Bøe K.E. (2004). Aggression and group size in domesticated pigs, Sus scrofa: when the winner takes it all and the loser is standing small. Anim. Behav..

[35] Büttner K., Scheffler K., Czycholl I., Krieter J. (2015). Social network analysis—centrality parameters and individual network positions of agonistic behavior in pigs over three different age levels. Springerplus.

[36] Rault J.-L., Lay D.C., Marchant-Forde J.N. (2011). Castration induced pain in pigs and other livestock. Appl. Anim. Behav. Sci..

[37] Shizuka D., McDonald D.B. (2012). A social network perspective on measurements of dominance hierarchies. Anim. Behav..

[38] Hessing M.J.C., Hagelsø A.M., Schouten W.G.P., Wiepkema P.R., van Beek J.A.M. (1994). Individual Behavioral and Physiological Strategies in Pigs. Physiol. Behav..

[39] Hessing M.J.C., Hagelsø A.M., van Beek J.A.M., Wiepkema P.R., Schouten W.G.P., Krukow R. (1993). Individual behavioural characteristics in pigs. Appl. Anim. Behav. Sci..

[40] Arey D.S., Franklin M.F. (1995). Effects of straw and unfamiliarity on fighting between newly mixed growing pigs. Appl. Anim. Behav. Sci..

[41] Camerlink I., Arnott G., Farish M., Turner S.P. (2016). Complex contests and the influence of aggressiveness in pigs. Anim. Behav..

[42] Carter A.J., Feeney W.E., Marshall H.H., Cowlishaw G., Heinsohn R. (2013). Animal personality: What are behavioural ecologists measuring?. Biol. Rev..

[43] Meese G.B., Ewbank R. (1973). The establishment and nature of the dominance hierarchy in the domesticated pig. Anim. Behav..

[44] Coutellier L., Arnould C., Boissy A., Orgeur P., Prunier A., Veissier I., Meunier-Salaün M.-C. (2007). Pig’s responses to repeated social regrouping and relocation during the growing-finishing period. Appl. Anim. Behav. Sci..

[45] Mendl M., Zanella A.J., Broom D.M. (1992). Physiological and reproductive correlates of behavioural strategies in female domestic pigs. Anim. Behav..

